# Single-Incision Laparoscopic Surgery for Undiagnosed Small Bowel Obstruction in a Patient without a History of Abdominal Surgery

**DOI:** 10.1155/2015/942393

**Published:** 2015-03-16

**Authors:** Noriaki Koizumi, Hiroki Kobayashi, Tsuyoshi Takagi, Kanehisa Fukumoto

**Affiliations:** Department of Surgery, Nishijin Hospital, 1035 Mizomae-cho, Kamigyo-ku, Kyoto 602-8319, Japan

## Abstract

We herein report a 66-year-old female patient who developed an undiagnosed small bowel obstruction without a history of prior abdominal surgery and was successfully treated by single-incision laparoscopic surgery. A small bowel obstruction with unknown cause typically requires some sort of surgical treatment in parallel with a definitive diagnosis. Although open abdominal surgery has been generally performed for the treatment of small bowel obstructions, laparoscopic surgery for small bowel obstructions has been increasing in popularity due to its less invasiveness, including fewer postoperative complications and a shorter hospital stay. As a much less invasive therapeutic strategy, we have performed single-incision laparoscopic surgery for the treatment of an undiagnosed small bowel obstruction. We were able to make a definitive diagnosis after sufficient intra-abdominal inspection and to perform enterotomy through a small umbilical incision. Single-incision laparoscopic surgery appears to be comparable to conventional laparoscopic surgery and provides improved cosmesis, although it is an optional strategy only applicable to selected patients.

## 1. Introduction

Although a small bowel obstruction develops most frequently as a result of postoperative adhesions after abdominal surgery, cases which developed in a patient without a history of prior abdominal surgery have been sometimes reported [1, 2]. While most small bowel obstructions require some sort of surgical treatment, laparoscopic surgery is becoming a promising option for the management of small bowel obstructions and is considered a reasonable approach because of its less invasiveness and improved short-term outcomes compared to open surgery [3, 4]. We herein present a rare case of an undiagnosed small bowel obstruction which developed in a patient without a history of prior abdominal surgery and was successfully treated by single-incision laparoscopic surgery (SILS).

## 2. Case Presentation

**Figure 1:**
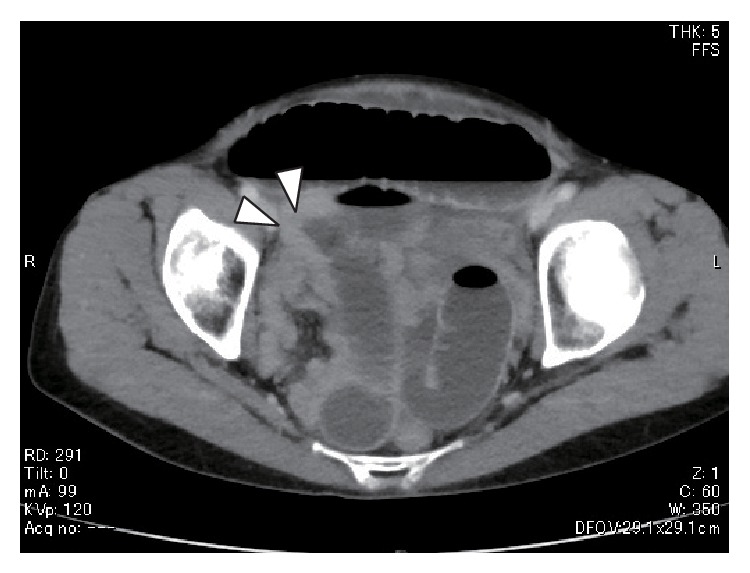
An abdominal computed tomography showed a small bowel dilatation with a caliber change (arrowhead).

**Figure 2:**
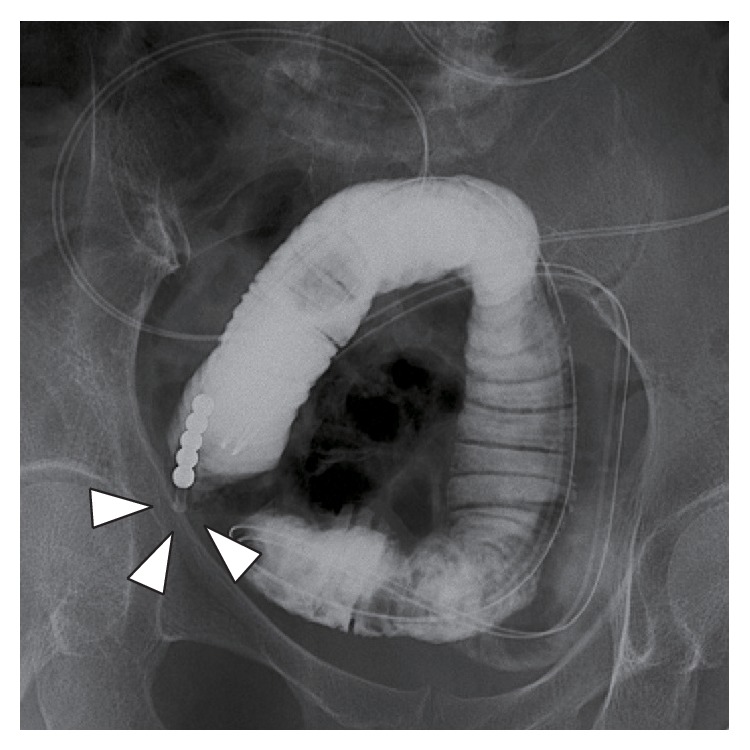
A contrast study showed a focal small bowel obstruction (arrowhead).

**Figure 3:**
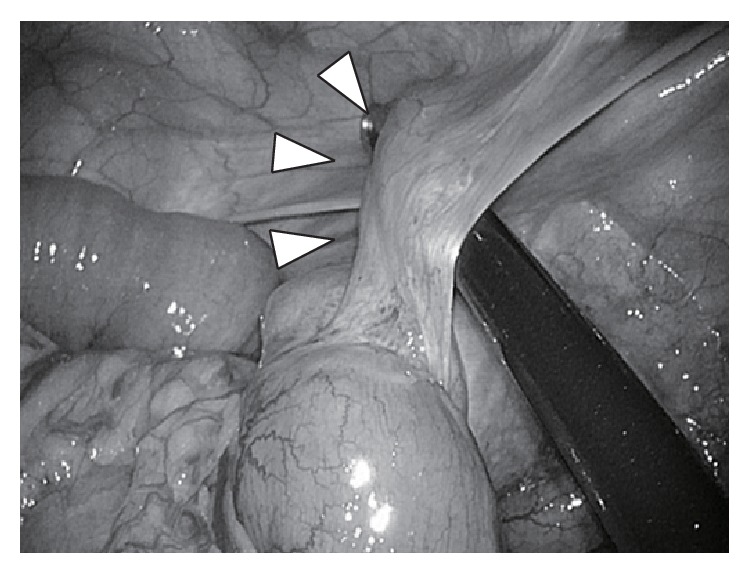
An intra-abdominal inspection showed an abnormal cord-like structure adhered to the ileum (arrowhead).

**Figure 4:**
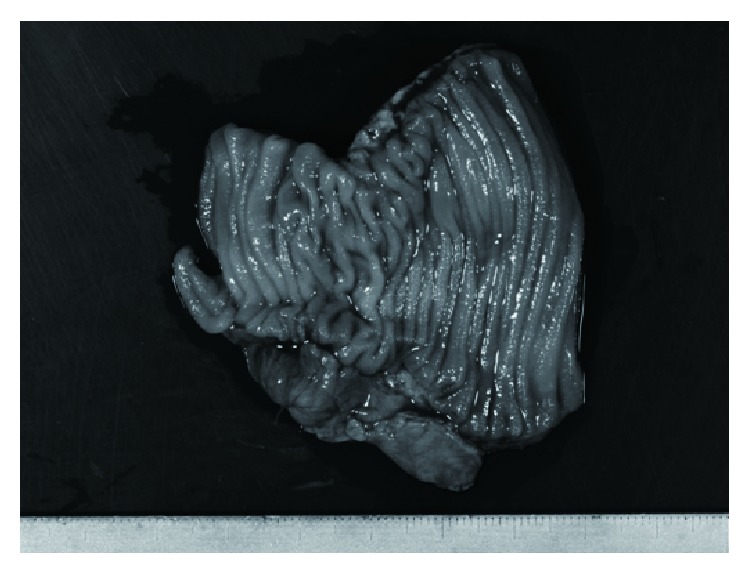
The excised ileum showed no particular abnormal findings despite the caliber change.

A 66-year-old female patient presented with vomiting, abdominal fullness, and constipation. She had no particular previous history except for a right inguinal hernia which had healed spontaneously during childhood. An abdominal computed tomography revealed diffuse small bowel dilatation with a caliber change at the right side of the pelvis (Figure 1). There was no apparent cause of obstruction, such as internal hernia, intestinal torsion, or a tumor. Therefore, she was diagnosed as having a small bowel obstruction with unknown cause and received a long intestinal tube insertion in order to decompress the dilated small bowel. Despite successful decompression, a contrast study showed persisting bowel obstruction (Figure 2). Since the cause of the focal small bowel obstruction was unknown, the patient underwent SILS both for a diagnosis and treatment. Under general anesthesia, a small incision at the umbilicus was made, and an access port (EZ ACCESS, Hakko. Co. Ltd., Nagano, Japan) equipped with three 5 mm trocars was set. Intra-abdominal inspection revealed an abnormal cord-like structure which was continuous to the peritoneum near the right inguinal region. No apparent inguinal hernia was observed. The cord-like structure closely adhered to the ileum, resulting in ileal stenosis (Figure 3). It was then cut by the ultrasonic coagulating shears, and the ileum was pulled out extracorporeally from the umbilical small incision. Since the diseased ileum showed apparent caliber change around the site of adhesion, we performed partial resection of the ileum, including the cord-like structure, and the ileum was reconstructed by functional end-to-end anastomosis using linear staplers. No abnormal findings were observed in the mucosa of the excised ileum other than an apparent caliber change (Figure 4). Although the histopathological examination revealed spindle cell proliferation in the subserosal area of the ileum, there was no evidence of a neoplastic change. The patient recovered without any complications and was discharged 8 days after surgery. She has remained healthy with no recurrence of small bowel obstruction for 1 year.

## 3. Discussion

Although most small bowel obstructions are caused by the presence of adhesions after abdominal surgery, there are a few cases that develop without a history of prior abdominal surgery. Among such cases, the major causes are reported to include Crohn's disease, neoplasia, hernia, and radiation, while the etiology of about 10% is miscellaneous [2, 5]. Particularly, the incidence rate for unexplained adhesional small bowel obstruction in the absence of prior abdominal surgery is exceptionally low with a reported incidence of only 3.3% [1]. Since such small bowel obstructions are difficult to diagnose and tend to be intractable to conservative treatments, most of them require some sort of surgical treatment in parallel with a definitive diagnosis.

With the recent technical advancement of laparoscopic surgery, the laparoscopic approach for the treatment of small bowel obstructions has become increasingly popular because of less invasiveness, including fewer postoperative complications and a shorter hospital stay [4, 6]. Since open abdominal surgery may trigger additional intra-abdominal adhesions, laparoscopic surgery with a negligible abdominal incision is considered to be helpful for the prevention of recurrent adhesional small bowel obstruction.

While conventional laparoscopic surgery has a low level of invasiveness, SILS is now widely performed as a much less invasive therapeutic procedure in many fields. In fact, SILS is also performed for the treatment of a portion of small bowel obstructions at limited institutions, although it is still considered to be an optional strategy only applicable to selected patients with small bowel obstructions [7, 8].

In the surgical treatment of small bowel obstructions, enterotomy is sometimes required when ischemic change, stenosis, or neoplasia is observed. In such cases, we can pull out the diseased small intestine extracorporeally through a small umbilical incision during the SILS procedure. In addition, we can also extract the resected specimen through a minimal incision of the umbilicus, even when the diseased lesion is intracorporeally resected. Thus, the small intestine is considered to be a suitable organ for SILS.

SILS is also advantageous in that it can minimize postoperative adhesion and port-related complications and also results in improved cosmesis. Although a dedicated access port is needed, SILS appears to have no particular disadvantages to the conventional laparoscopic procedure in selected patients with small bowel obstruction.

In our case, the pathogenesis of the cord-like structure was undetermined even after performing a detailed pathological inspection. Although no previous report describing the association between spontaneous healing of inguinal hernia and formation of following abnormal adhesion has been published, the potential involvement of prior spontaneously healed inguinal hernia has been suggested.

In conclusion, we concurrently achieved a definitive diagnosis and then performed appropriate treatment with minimal invasiveness through the SILS procedure. SILS therefore appears to be a suitable approach for the treatment of small bowel obstruction of unknown cause and it is considered to be a safe and promising treatment option. However, we should note that its application is limited to only selected patients and it does not completely overwhelm conventional laparoscopic surgery.
